# Prevalence, Pattern, Mortality, and Morbidity of Traumatic Small Bowel Perforation at King Abdulaziz Medical City: A Retrospective Cohort Study

**DOI:** 10.7759/cureus.52313

**Published:** 2024-01-15

**Authors:** Fahad Aljehaiman, Faisal J Almalki, Abdulah Alhusain, Faris Alsalamah, Khaled Alzahrani, Abdulkareem Alharbi, Hani Alkhulaiwi

**Affiliations:** 1 College of Medicine, King Saud Bin Abdulaziz University for Health Sciences, Riyadh, SAU; 2 Research, King Abdullah International Medical Research Center, Riyadh, SAU; 3 Plastic and Reconstructive Surgery, King Abdulaziz Medical City, Riyadh, SAU; 4 Preventive Medicine, King Abdulaziz Medical City, Riyadh, SAU; 5 Trauma and Acute Care Surgery, King Abdulaziz Medical City, Riyadh, SAU

**Keywords:** motor vehicle collision, penetrating abdominal injury, blunt abdominal injury, bowel injury, traumatic small bowel perforation

## Abstract

Introduction

Bowel perforation, whether from trauma or other causes, presents with diverse clinical scenarios. Small bowel perforation (SBP), a potentially fatal condition often linked to blunt trauma like motor vehicle accidents, necessitates prompt detection and intervention, crucial for improved outcomes. This study investigated the prevalence, predictors, presentation, diagnostic findings, morbidity, and mortality of traumatic SBP for comprehensive insights.

Methodology

This was a retrospective cohort study conducted at King Abdulaziz Medical City, Riyadh. A review of 838 cases, which represent all abdominal trauma patients from January 2017 to March 2023, was done. Forty patients who developed SBP and have complete data were included in this study. One case was excluded due to incomplete medical records. Data were collected with the non-probability convenience sampling technique via the BestCare system using a data collection sheet. Data were analyzed with IBM SPSS 29 (IBM Corp., Armonk, NY).

Results

Out of all abdominal trauma cases (n=838), 40 patients developed SBP (n=40, 4.77%). Males constituted 87.5%, and the most common mechanism was motor vehicle accidents (57.5%). Complications included cardiac arrest, disseminated intravascular coagulation (DIC), and leak (7.5% each). In motor vehicle accidents, SBP primarily affected patients who were in the driver's position (78.3%). Clinical signs at presentation revealed abdominal tenderness (52.5%), abdominal distension (22.5%), and abnormal systolic blood pressure (mean 115.3 mmHg). Linear regression showed gender and age positively associated with morbidity (p=0.474, p=0.543) while BMI exhibited a negative relationship (p=0.314). Logistic regression revealed non-significant predictors of mortality, except for mean initial hematocrit (HCT) (p=0.721, aOR=0.098).

Conclusion

Our study provides crucial findings on the incidence, patterns, mortality, and morbidity of traumatic bowel perforation, contributing to the existing body of research. The identified prevalence of 4.77% and mortality at 17.5% from the studied population underline the serious impact of this condition, and the 37.5% complication rate observed demonstrates the potential risks involved. The average hospital stay is found to be 14 days, adding further to the disease burden. These findings underscore the importance of specific preventative measures, particularly related to motor vehicle accidents (MVAs), and highlight potential markers for predicting outcomes, such as age, gender, and mean initial HCT. This substantiates the need for further research involving larger cohorts and prospective designs to gain comprehensive insights and establish more robust preventative and treatment strategies.

## Introduction

Bowel perforation, also known as intestinal perforation, is a condition that involves local damage to the integrity and continuity of the bowel wall. Bowel perforation can be of two types: free or contained perforation. Free (open) perforations are those in which an open hole is created allowing the intestinal material to escape into the normally sterile peritoneal cavity and can cause infection, inflammation, and other complications to the surrounding tissues and organs. On the other hand, contained (closed) perforations are those in which a full-thickness hole is created by an ulcer, but it is sealed by the adjacent organs and free spillage is prevented [1]. In general, the clinical presentation of small bowel perforation (SBP) is highly diverse and depends on the underlying cause, type of perforation, and site. SBP can present acutely with symptoms ranging from localized abdominal pain to systemic symptoms like fever, nausea, vomiting, and even shock [1]. However, there are rising concerns due to several reported cases that highlighted delayed small bowel perforations following blunt trauma [2].

The etiologies behind small bowel perforation are various and include traumatic, iatrogenic, and non-traumatic causes (such as inflammatory conditions, infections, ischemic changes, diverticula, and foreign bodies) [3]. Traumatic causes of bowel perforation can be due to blunt or penetrating injuries. The nature of the small intestines being coiled and occupying the largest portion of the abdominal cavity makes them the most injured intra-abdominal hollow organ, particularly from penetrating injuries such as knife stabbing wounds or gunshots [4]. Blunt traumas due to MVAs are one of the most common causes of SBP, especially in seat-belt users accounting for approximately 75% of gastrointestinal (GIT) trauma with potential perforation. Seat-belt syndrome is the range of injuries involving not only intra-abdominal injuries but also bone injuries mainly as a result of sudden deceleration of the abdominal organs due to seat-belt constraining them [5].

The diagnosis of SBP is usually made by imaging techniques that detect the presence of fluid or gases in the peritoneum or mediastinum (pneumoperitoneum and pneumomediastinum, respectively). A CT scan is regarded as the imaging modality of choice for hemodynamically stable patients; however, negative results don’t rule out the diagnosis of SBP. In the EAST trial, 13% of the study sample who were discovered to have an SBP at laparotomy had no positive findings prior to surgery [6]. Although erect plain radiographs are usually the first imaging modality to start with and are able to detect signs of perforation, they lack the ability to localize the site of perforation. Other diagnostic methods include ultrasonography and MRI, but previous studies demonstrated no substantial advantage over CT scans [3].

Although small bowel perforation is rare, it is a clinically complex medical condition with high mortality and should be diagnosed and managed early for a better prognosis [7,8]. SBP is usually managed by early stabilization, antibiotic therapy, and surgical source control depending on the case and the presence of strong indicators for prompt operative intervention [9-11]. The mortality rates among patients diagnosed with GIT trauma have been found to be linked to the delay in initial diagnosis, development of multi-organ failure, and sepsis as emphasized by the EAST study, which reported a rise in mortality rate from 4% to 16% when the diagnosis is delayed for more than 24h. it was further highlighted that when SBPs were present the mortality rate rose from 14% to 19% [6]. Unfortunately, significant gaps remain in existing knowledge and research data. For instance, the specific prevalence and mortality rates, especially in the context of temporal changes and regional variability, are not well-established. Additionally, the potential improvements in diagnosis, including the limitations of imaging techniques, such as CT scans, are not thoroughly researched.

Furthermore, while there's a broad understanding of the etiologies behind small bowel perforation and their implications, particularly traumatic causes, such as blunt or penetrating injuries detailed knowledge of their actual prevalence, and their patterns in specific contexts like MVAs is limited. This lack of data limits our ability to understand the full implications of these etiologies, hindering effective prevention and response efforts. Similarly, there's also a dearth of research papers characterizing the most frequent patterns, clinical presentations, diagnostic findings, and mechanisms of injury associated with traumatic bowel perforation, especially on an international scale, and in specific localities. The objective of this study is to identify the most common trends, clinical signs at presentation, diagnostic outcomes, and causes of injury associated with traumatic small bowel perforation at King Abdulaziz Medical City. Moreover, it endeavors to calculate the prevalence, mortality, and complication rates of this condition.

## Materials and methods

This research involved the receipt of ethics approval from the King Abdullah International Medical Research Center’s (KAIMRC) Institutional Review Board preceding commencement (approval number: IRB/2772/23). It was held at the King Abdulaziz Medical City in Riyadh (KAMC-RD), a tertiary National Guard hospital established by royal decree in May 1983, with provision for over 2100 beds. A comprehensive review of all medical records pertaining to abdominal trauma patients who displayed symptoms of small bowel perforation from January 2017 through to March 2023 was carried out. Patients with isolated small bowel perforation and concomitant small and large bowel perforations were included in this study. However, patients with incomplete records or isolated large bowel perforation were procedurally disqualified from inclusion in the research study. 

The study reviewed an expansive caseload of 838 abdominal trauma patients. Of these, 40 patients who developed small bowel perforation (SBP) and possessed comprehensive records were selected. The research employed a retrospective cohort study design using non-probability consecutive sampling [12].

Data collection was conducted using the BESTcare system, a product by the Saudi-Korean Health Informatics Company. The BESTcare system provided a methodological approach for organizing patient data and demographic information. As per research protocol, only researchers were involved in collecting and securely storing patient data, with all identification and personal details substituted with serial numbers to uphold privacy. Given the retrospective design of this study, informed consent was acknowledged since the study poses minimal risk and does not involve the collection of identifiable personal health information.

The data parameters included the patient demographics (age, gender, and BMI), type (penetrating or blunt trauma) of inflicted injury (such as gunshots, stab wound, MVA, and others), operative records, including type, and surgical results, including site of small bowel perforation, duration of hospital stay, and other co-morbid factors. For motor vehicle accident (MVA) cases, data regarding seat location, airbag deployment, seatbelt use, and presence of abdominal seatbelt marks upon initial inspection were documented, along with findings from diagnostic tests, such as ultrasound and CT scans, physical examination, initial vital signs, and lab results (WBC, HCT, and amylase).

The data were collected, and reviewed, and then statistical analysis was carried out using both descriptive and inferential models, with IBM's SPSS software (version 29.0.0; IBM Corp., Armonk, NY, USA) utilized for analysis. The descriptive statistical analysis provided an initial overview of the demographic characteristics (age, gender, etc.) of the patient population. Mean and standard deviation (SD) were used for quantitative variables while qualitative data were expressed as frequencies and percentages. Short and long-term complications of SBP were plotted in a figure. Further inferential analyses, including logistic and linear regression models, were utilized as predictive tools for evaluating mortality and morbidity outcomes in perforated bowel patients. The threshold for statistical significance was set at a p-value of less than or equal to 0.05 with a numeric 95% confidence interval.

## Results

Our study included 40 participants as shown in Table 1. Regarding gender, the majority were male, constituting 87.5% (n=35), while females comprised 12.5% (n=5). Regarding age distribution, the highest proportion fell within the 25-50 years category, representing 50% (n=20). Body mass index (BMI) classification indicated that 55% (n=22) of participants were of normal weight while 15% (n=6) were overweight, 17.5% (n=7) were obese, and the remaining 12.5% (n=5) were underweight. In terms of injury type, 75% (n=30) experienced blunt trauma, with the remaining 25% (n=10) having penetrating injuries. The most common mechanism of injury (MOI) was MVAs at 57.5% (n=23).

**Table 1:** Sociodemographic and injury features of participants (n=40) n: frequency; %: percentage; BMI: body mass index

Patients' Demographics		Frequency n(%)
			Gender	Female	5 (12.5)
Male	35 (87.5)		
Age	< 25 Years	12 (30.0)
	25-50 Years	20 (50.0)
	> 50 Years	8 (20.0)
BMI Class	Underweight	5 (12.5)
	Normal	22 (55.0)
	Overweight	6 (15.0)
	Obese	7 (17.5)
Injury Features		
Type of Injury	Blunt	30 (75.0)
	Penetrating	10 (25.0)
Mechanism of Injury	Motor vehicle accident	23 (57.5)
	Pedestrian struck	6 (15.0)
	Gunshot	5 (12.5)
	Stab wound	2 (5.0)
	Grass cutter	1 (2.5)
	Hit by a hoarse	1 (2.5)
	Nail gun	1 (2.5)
	Explosion	1 (2.5)

**Figure 1:**
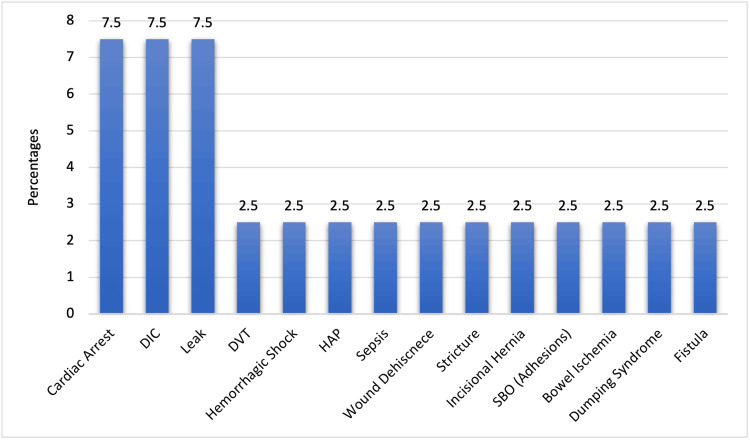
Different short and long-term complications of participants (n=40) DIC: disseminated intravascular coagulation; DVT: deep vein thrombosis; HAP: hospital-acquired pneumonia; SBO: small bowel obstruction

Figure 1 shows a breakdown of short and long-term complications observed among participants with traumatic bowel perforation. Cardiac arrest, disseminated intravascular coagulation (DIC), and leak each accounted for 7.5% of cases. Other complications, such as deep vein thrombosis (DVT), hemorrhagic shock, hospital-acquired pneumonia (HAP), sepsis, wound dehiscence, stricture, incisional hernia, small bowel obstruction (SBO) due to adhesions, bowel ischemia, dumping syndrome, and fistula, were each reported in 2.5% of cases.

Table 2 shows the features and patterns of injury associated with MVAs. The majority of injuries occurred in the driver position, constituting 78.3% (n=18), while 21.7% (n=5) were in non-driver positions. The deployment status of airbags was predominantly unknown in 73.9% (n=17) of cases, with 21.7% (n=5) indicating no deployment and only 4.3% (n=1) reporting airbag deployment. Seatbelt use was often unrecorded (17.4%, n=4), but when documented, 47.9% (n=11) indicated no seatbelt use and 34.7% (n=8) reported usage. Notably, abdominal seatbelt marks were present in 21.8% (n=5) of SBP cases.

**Table 2:** Different features and patterns of injury related to motor vehicle accidents (n=23) n: frequency; %: percentage; MVA: motor vehicle accident

MVA-Related Features and Patterns		Frequency n(%)
			Seat Position	Non-Driver Position	5 (21.7)
Driver Position	18 (78.3)		
Deployment of Air Bag	Unknown	17 (73.9)
	Not Deployed	5 (21.7)
	Deployed	1 (4.3)
Seatbelt Use	Unknown	4 (17.4)
	No	11 (47.9)
	Yes	8 (34.7)
Abdominal Seatbelt Mark	No	18 (78.2)
	Yes	5 (21.8)

Table 3 shows a comprehensive overview of clinical signs, investigations, and interventions related to perforated bowel injury after trauma. Abdominal tenderness was present in 52.5% (n=21) of cases while abdominal distension was reported in 22.5% (n=9) and 17.5% (n=7) showed peritoneal signs. Systolic blood pressure had a mean value of 115.3 mmHg (SD 34.8), with a wide range from 0 to 177 mmHg. Imaging findings revealed that the majority of chest X-rays were normal (85%, n=34), and abdominal X-rays were mostly not done (92.5%, n=37). Abdominal ultrasound identified positive findings of free intra-abdominal fluid in 55% (n=22) of cases. The findings from CT scan evaluations of the participants are as follows. Only three patients (7.5%) presented with normal CT scan findings. The most common CT scan finding was free air/fluid, observed in 21 patients accounting for 52.5% of the sample size. Fat stranding and Ischemic bowel were seen in four patients each, constituting 10% of the study population respectively. Pneumatosis intestinalis was observed in two patients, making up 5% of the sample size. Furthermore, a relatively rare finding, a chance fracture, was seen in a single patient accounting for 2.5% of the total participants. Regarding lab findings, the mean initial white blood cell (WBC) level was 15.1 (SD 7.1), with a broad range from 4.9 to 35.6. The mean percentage of the initial WBC level was 40 x10^9^/L (SD 8.2), ranging from 15.7 to 51.2 x10^9^/L. The mean initial amylase level was 77.2 U/L (SD 43.1), with a range from 31 to 213 U/L. Among the participants, 25% (10/40) underwent diagnostic laparoscopy, which was converted to laparotomy in all cases. Direct exploratory laparotomy was performed in 62.5% (25/40) of cases while 12.5% (5/40) were managed conservatively without surgical intervention. Out of those managed surgically (n=35), 74.28% (26/35) underwent resection of the perforated segment and anastomosis while 25.72% (9/35) underwent primary repair of the perorated segment.

**Table 3:** Clinical signs, investigations, and interventions related to perforated small bowel injury after trauma (n=40) n: frequency; %: percentage; SD: standard deviation; BP: blood pressure; US: ultrasound; CT: computed tomography; WBC: white blood cell count; HCT: hematocrit; mmHG: millimeters of mercury; SBP: small bowel perforation

Clinical signs		Frequency n (%)
Abdominal Tenderness	No	19 (47.5)
	Yes	21 (52.5)
Abdominal Distension	No	31 (77.5)
	Yes	9 (22.5)
Peritoneal Signs	No	33 (82.5)
	Yes	7 (17.5)
Systolic BP (mmHg)	Mean (SD)	115.3 (34.8)
	Range	0-177
Imaging Findings		
Chest X-Ray Findings	Unremarkable	34 (85)
	Rib Fractures	2 (5.0)
	Clavicular Fracture	2 (5.0)
	Pulmonary Contusions	1 (2.5)
Abdominal X-Ray Findings	Normal	3 (7.5)
	N/A	37 (92.5)
Abdominal USG Findings	Negative	15 (37.5)
	Positive	22 (55.0)
	N/A	3 (7.5)
CT Scan Findings	Normal	3 (7.5)
	N/A	6 (15%)
	Free Air/Fluid	21 (52.5)
	Fat stranding	4 (10.0)
	Ischemic bowel	4 (10.0)
	Pneumatosis intestinalis	2 (5.0)
	Chance fracture	1 (2.5)
Lab Findings		
Initial WBC Level (x10^9^/L)	Mean (SD)	15.1 (7.1)
	Range	4.9-35.6
Initial HCT Level (%)	Mean (SD)	0.4 (0.08)
	Range	0.15-0.51
Initial Amylase (U/L)	Mean (SD)	77.2 (43.1)
	Range	31-213
Intervention and Management		
Intervention	Diagnostic Laparoscopy Converted to Laparotomy	10 (25)
	Direct Exploratory Laparotomy	25 (62.5)
	Conservative	5 (12.5)
Surgical Intervention for SBP	Resection and Anastomosis	26 (65)
	Primary Repair	9 (22.5)

Table 4 shows the intra-operative findings and the state of intra-abdominal organs in traumatic small bowel perforation patients. Regarding the type of perforation, 62.5% (25) of the cases had a single SBP, 27.5% (11) had multiple SBPs, and 10% (4) had concomitant SBP/LBP. In terms of the state of the mesentery, 5% (2) of patients exhibited oozing/bleeding, 12.5% (5) had a hematoma, and 40% (16) had a laceration. Other findings included Leeser sac hematoma (2.5%, 1 case), bucket handle injury (12.5%, 5 cases), retroperitoneal hematoma (5%, 2 cases), and serosal tear (12.5%, 5 cases). Regarding the state of intra-abdominal organs, spleen injuries were observed in 10 patients. The distribution of spleen injuries by grade was as follows: Grade 1 (5%, 2 cases), Grade 2 (5%, 2 cases), Grade 3 (10%, 4 cases), Grade 4 (2.5%, 1 case), and Grade 5 (2.5%, 1 case). Renal injuries were observed in 6 patients, with Grade 1, Grade 2, and Grade 3 injuries each accounting for 5% of the cases. Liver injuries were found in five patients, with Grade 2 injuries accounting for 7.5% (3 cases) and Grade 3 injuries accounting for 5% (2 cases). Stomach injuries were observed in four patients, with gastric antrum contusion accounting for 2.5% (1 case) and perforation accounting for 7.5% (3 cases). Adrenal injuries were observed in four patients, all of whom presented with hematomas. Additionally, two cases of pancreatic injury were reported. The specific sites of perforation were investigated. Among the cases with the perforation located in the jejunum, a total of 15 patients were included. The range of the jejunum perforation sites was 20-170 cm away from the duodenojejunal junction, with a mean distance of 48.33 cm (SD: 47.3). In cases where the perforation was located in the ileum, a total of 11 patients were included. The range of the ileum perforation sites was 10-250 cm proximal to the ileocecal valve, with a mean distance of 124.54 cm (SD: 96.47). No specific data on the site of perforation were available for 8 cases involving the jejunum/ileum.

**Table 4:** Intra-operative findings and state of intra-abdominal organs in traumatic SBP patients n: frequency; %: percentage; SBP: small bowel perforation; LBP: large bowel perforation; DJ: duodenojejunal junction; IC: ileocecal valve

Intra-Operative Findings			n (%)
Type of Perforation	Single SBP		25 (62.5)
	Multiple SBPs		11 (27.5)
	Concomitant SBP/LBP		4 (10)
State of Mesentery	Oozing / Bleeding		2 (5)
	Hematoma		5 (12.5)
	Laceration		16 (40)
Other Findings	Leeser sac hematoma		1 (2.5)
	Bucket handle injury		5 (12.5)
	Retroperitoneal hematoma		2 (5)
	Serosal tear		5 (12.5)
State of Intra-Abdominal Organs			
Spleen Injury (n=10)	Grade 1		2 (5)
	Grade 2		2 (5)
	Grade 3		4 (10)
	Grade 4		1 (2.5)
	Grade 5		1 (2.5)
Renal Injury (n=6)	Grade 1		2 (5)
	Grade 2		2 (5)
	Grade 3		2 (5)
Liver Injury (n= 5)	Grade 2		3 (7.5)
	Grade 3		2 (5)
Stomach Injury (n= 4)	Gastric antrum contusion		1 (2.5)
	Perforation		3 (7.5)
Adrenal Injury (n=4)	Hematoma		4 (10)
Pancreatic Injury			2 (5)
The Specific Site of Perforation			
Jejunum (away from DJ junction) n=15	Range	20-170 cm	
	Mean (SD)	48.33 cm (47.3)	
Ileum (proximal to IC valve) n=11	Range	10-250 cm	
	Mean (SD)	124.54 cm (96.47)	
Jejunum/Ileum (n=8)	N/A		

Table 5 shows the results from a linear regression model on predictors of morbidity in perforated bowel patients. Gender and age exhibited positive associations with morbidity (B = 109.999, Beta = 0.452, p = 0.474; B = 2.914, Beta = 0.350, p = 0.543), suggesting increased morbidity for male and older individuals. Conversely, BMI displayed a negative relationship (B = -10.284, Beta = -0.505, p = 0.314), indicating lower morbidity with higher BMI. Other predictors, including MVA, initial WBC level, initial HCT level, initial amylase level, and systolic BP, lacked statistically significant associations with morbidity. However, all these variables are non-significant predictors of morbidity in perforated bowel patients.

**Table 5:** Predictors of morbidity in perforated small bowel patients (linear regression model) CI: confidence interval; BMI: body mass index; MVA: motor vehicle accident; WBC: white blood cell count; HCT: hematocrit; BP: blood pressure

Predictors of Morbidity	Unstandardized Coefficients		Standardized Coefficients	Significance	95% CI	
	B	Standard Error	Beta		Lower Bound	Upper Bound
Gender (Male)	109.999	145.276	.452	.474	-233.524	453.522
Age	2.914	4.556	.350	.543	-7.859	13.687
BMI	-10.284	9.482	-.505	.314	-32.704	12.137
MVA	-26.841	99.961	-.126	.796	-263.212	209.530
Initial WBC Level	-4.333	6.804	-.285	.544	-20.423	11.756
Initial HCT Level	-380.018	814.519	-.238	.655	-2306.049	1546.012
Initial Amylase Level	-.389	1.034	-.159	.718	-2.834	2.056
Systolic BP	-1.009	2.844	-.213	.733	-7.735	5.717

Table 6 shows the predictors of mortality in patients with perforated bowel injuries. For gender, the coefficient (B = -0.527, p = 0.773) demonstrated a non-significant negative association, resulting in an adjusted odds ratio (aOR) of 0.590 (95% CI: 0.017-21.103). Age showed a non-significant positive association (B = 0.058, p = 0.108), yielding an aOR of 1.060 (95% CI: 0.987-1.137). BMI had a non-significant positive association (B = 0.139, p = 0.348), producing an aOR of 1.149 (95% CI: 0.860-1.535). MVA, systolic BP, and mean initial WBC did not significantly predict morbidity. However, the mean initial HCT exhibited a highly significant negative association (B = -2.328, p = 0.721), resulting in an aOR of 0.098 (95% CI: 0.000-35004.598).

**Table 6:** Predictors of mortality in perforated small bowel patients (logistic regression model) aOR: adjusted odds ratio; CI: confidence interval; BMI: body mass index; MVA: motor vehicle accident; BP: blood pressure; WBC: white blood cell count; HCT: hematocrit

Predictors of Mortality	B	Sig.	aOR	95% CI	
				Lower	Upper
Gender	-.527	.773	.590	.017	21.103
Age	.058	.108	1.060	.987	1.137
BMI	.139	.348	1.149	.860	1.535
MVA	-.542	.704	.582	.035	9.563
Systolic BP	-.028	.133	.973	.938	1.009
Initial WBC	-.062	.515	.940	.780	1.133
Initial HCT	-2.328	.721	.098	.000	35004.598
Constant	-2.031	.668	.131		

Table 7 shows the timing of presentation, length of hospital stay, morbidity, and mortality among study cases with small bowel perforation, King Abdulaziz Medical City. For timing of presentation, a total of 33 (82.5%) cases were in ER directly but two cases were delayed (after 8 hours or more). A total of 15 (37.5%) cases had complications that are presented in Figure 1. Considering mortality, seven (17.5%) cases expired after developing SBP. The median length of hospital stay was 14 days. Among the patients, 45% (18) required postoperative admission to the intensive care unit (ICU) while 55% (22) did not require ICU admission. Regarding intra-abdominal drain insertion, 60% (24) of patients had an intra-abdominal drain inserted, whereas 40% (16) did not require drain insertion.

**Table 7:** Morbidity and mortality among study cases with small bowel perforation, King Abdulaziz Medical City n: frequency; %: percentage; ER: emergency department; ICU: intensive care unit

Morbidity and Mortality	n	%
Timing of presentation		
Immediate to ER	33	82.50%
Delayed	2	5.00%
Unknown	5	12.50%
Complications		
Yes	15	37.50%
No	25	62.50%
Need for ICU admission postoperatively		
Yes	18	45.00%
No	22	55.00%
Intra-abdominal drain insertion		
Yes	24	60.00%
No	16	40.00%
Length of hospital stay		
< 1 week	12	30.00%
1-2 weeks	9	22.50%
2 weeks-1 month	12	30.00%
> 1 month	7	17.50%
Range (median)	3 hrs-455 (14 days)	
Mortality		
Died	7	17.50%
Survived	33	82.50%

## Discussion

The originality and significance of our study lie in its comprehensive analysis of traumatic small bowel perforation epidemiology, patterns, mortality, and morbidity among 40 largely male participants aged 25-50 years, most with a normal weight BMI, and subjected predominantly to blunt trauma injuries induced mainly by motor vehicle accidents. Presenting one of the first detailed demographic and clinical profiles of traumatic small bowel perforation.

Bowel perforation, whether free or contained, poses serious health risks, with diverse clinical presentations. Small bowel perforation (SBP) results from traumatic, iatrogenic, or non-traumatic causes [13,14]. Blunt traumas, especially from motor vehicle accidents, are common, causing SBP with potential seat-belt injuries. Sule et al. (2007) show that peritonitis following a bowel perforation after blunt abdominal trauma is often present at the time of presentation and diagnosis is usually made [15]. Diagnosis relies on imaging, mainly CT scans, though negative results do not exclude SBP. Early detection and management are crucial for a better prognosis, involving stabilization, antibiotics, and surgical intervention. Limited research exists on traumatic SBP trends, prompting this study to investigate predictors, prevalence, and management outcomes. Our study aimed to investigate various aspects of traumatic bowel perforation, including prevalence, patterns, mortality, morbidity, and associated factors. Thus, we will analyze and contextualize our findings in comparison with existing medical literature.

Regarding gender, our results (Table 1) show that there is a higher prevalence of traumatic bowel perforation in males (87.5%), which is comparable to the results of a study by Onken et al. (2022), which indicated that 50.2% of participants with small bowel perforation were males while 49.8% of participants were females [7]. Our results are also comparable to the findings of a study by Mukhopadhyay et al. (2009), which showed that the male-to-female ratio for traumatic bowel injury from blunt abdominal trauma was 8.4: 1 [16]. Our study reveals that the age distribution indicated a peak incidence in the 25-50 years age group (50%), reflecting the age group most commonly exposed to traumatic events. This finding is consistent with the results of a study by Mohamed et al. (2023), which found that among different age groups, the 18-25 age group had the highest rate of road traffic accidents (RTAs) (11.89%), followed by the 26-35 age group (2.7%) [17].

The predominant mechanism of injury being MVA at 57.5% is in concordance with literature emphasizing the role of road traffic accidents in abdominal trauma. O'Rourke et al. (2023) showed that blunt abdominal trauma, prevalent in adults and children, often stems from motor vehicle accidents, constituting a common emergency room presentation [18]. Another study by Durrant et al. (2020), found that bowel injury often resulted from motor vehicle accidents, with 70%-85% of cases being blunt abdominal trauma [19].

Traumatic bowel perforation can culminate in a variety of short and long-term complications, illustrating the complex nature of this condition's outcomes. Significant complications such as cardiac arrest, DIC, and leak are noted, reaffirming the severe and systemic consequences of this injury. It's important to note that DIC in this context can occur due to a fibrinolytic phenotype triggered by the trauma and may be exacerbated by accompanying conditions such as anemia, hemodilution, hypothermia, and acidosis. These results are comparable to the findings of a study by O'Rourke et al. (2023), which indicated that sepsis, or septic shock, which is a recognized consequence of traumatic bowel perforation, could contribute to the development of DIC and potentially progress to multi-organ failure and cardiac arrest [18]. Jones et al. (2022) also support our results by showing that traumatic bowel perforation may cause other complications such as hernias, fistula formation, postoperative adhesions, hemodynamic instability, and delayed wound healing. Hemodynamic instability may result in other complications such as infection, multi-organ system failure, and hypoperfusion [14]. Beyond these well-documented complications, our study uncovers less frequent occurrences like Dumping syndrome and fistula, broadening the known spectrum of potential outcomes. The exploration of these complications aligns with existing literature and provides additional insights to complement the findings of other studies.

Moreover, the injury patterns related to MVAs, demonstrated that the driver position and unknown airbag deployment were common features. The prevalence of seatbelt non-usage in our study (47.9%) underscores the importance of educational campaigns promoting seatbelt adherence to prevent traumatic bowel injuries. This is consistent with the results of a study by Fouda Mbarga et al. (2018), which demonstrated that unbelted passengers had a higher risk of abdominal injuries and other injuries during MVAs compared to belted passengers [20]. Additionally, Fouda Mbarga et al. (2018) emphasized the significance of adhering to seatbelt usage to prevent traumatic bowel injuries [20]. The presence of abdominal seatbelt marks in 21.8% of cases is comparable to the findings of previous research by Biswas et al. (2014), emphasizing the role of seatbelts in both preventing and causing abdominal injuries [21]. Biswas et al. (2014) showed that patients with seatbelt injuries have solid organ injuries and require urgent intervention [21]. Bège et al. (2016) also argued that in 20% to 60% of cases, seatbelt marks were linked to intra-abdominal injury, which is comparable to our results [22]. The incidence of perforated bowel injury is increased by a ratio of 2.4 when there is seat belt bruising.

The onset time of symptoms subsequent to trauma plays a substantial role in the clinical symptoms and medical handling of small bowel perforations. Acute presentation is often characterized by localized abdominal pain and systemic symptoms such as fever, nausea, vomiting, and occasionally shock. On the other hand, a delayed presentation, which happens hours or even days post-trauma, may show a gradual escalation of pain and discomfort supplemented by other systemic symptoms. Our study results from 40 patients indicate this variability, with two individuals presenting after a delay of more than eight hours post-trauma, which is consistent with the results of previous research by Jahroml et al. (2016) showcasing similar instances of delayed presentations of ileum or jejunum perforation following blunt abdominal trauma, based on English-language medical literature [2]. Jahroml et al. (2016) maintained that a delayed presentation of SBP is characterized by hemorrhage, transmural congestion, and mucosal necrosis, which is comparable to our findings [2].

Notably, various clinical signs and investigations associated with perforated bowel injuries. Abdominal tenderness and distension, systolic blood pressure values, and imaging findings were consistent with expectations for diagnosing traumatic bowel perforation. The laboratory findings, including the mean initial white blood cell (WBC) and amylase levels, were in line with the expected markers of systemic response to trauma. These findings are comparable to the results of a previous study by Jones et al. (2023), which indicated that perforated bowel injuries are characterized by tenderness or pain in the abdomen, vomiting and nausea, chills or fever, and swollen abdomen and bloating [14]. Additionally, rigidity, guarding, and other peritoneal signs may develop as the condition progresses.

Moreover, the linear regression model identifies the predictors of morbidity, indicating that gender and age positively correlated with morbidity while BMI exhibited a negative association. These findings align with the results of a study by Giustozzi et al. (2021), linking male gender to increased morbidity in abdominal trauma cases [23]. The negative association of BMI with morbidity may reflect the potential protective effect of higher BMI in these patients, which warrants further investigations. Our results are also comparable to the finding of Shin et al. (2016) that morbidity following an intestinal perforation was greater in patients with unstable initial vital signs, poor nutritional status, and feculent ascites [24].

Various predictors of mortality using logistic regression, demonstrated non-significant associations for gender, age, BMI, and other variables. The non-significant associations may be attributed to the relatively small sample size and the complex nature of traumatic bowel injuries. Notably, mean initial HCT showed a negative association, suggesting potential value as a predictor of survival. These findings are in line with Reddy et. (2018) finding that concurrent chemoradiotherapy, adjuvant or primary radiotherapy, operative intervention, previous surgery, BMI, and age did not have negative clinical outcomes regarding mortality [25]. However, Yan et al., (2023) show that advanced age, shock on admission, elevated serum creatinine levels, and significantly abnormal WBC count are associated with higher in-hospital mortality following emergency laparotomy, which is inconsistent with our findings [26].

Limitations

The retrospective design of our study is a significant limitation, making it dependent on historical data and thereby subject to potential data inaccuracies or omissions. This type of design also restricts our ability to elicit causal relationships and control for confounding variables. Additionally, the small sample size may not perfectly represent the broader population. This constraint limits the statistical power of the study, thus results may not be generalizable to larger, more diverse populations, which can limit the extent of inferences drawn.

Our study may be subject to selection bias because not all eligible patients during the study period might have been included, especially if their documentation was not readily accessible in the database system. Therefore, the cohort might not reflect all patients suffering from traumatic bowel perforation.

Furthermore, certain aspects of patient morbidity, such as duration of ICU admission if needed, discharge on drains, and the need for mechanical ventilation, which are important outcomes in the study of traumatic bowel perforation, were not accounted for because of missing data. The lack of these crucial details impairs our ability to comprehensively analyze and understand the impact, seriousness, and prognosis of the condition.

Future direction and implications

Considering the limitations identified in our study, the direction for future research should involve prospective study designs, increasing the sample size, reducing selection bias, and ensuring comprehensive data collection. Furthermore, the establishment of multicenter studies and the inclusion of additional outcome measures like quality of life, psychological impact, and long-term survival rates could provide a more holistic view of traumatic bowel perforation. The implementation of these measures will be instrumental in filling current knowledge gaps and contribute to a more integrated understanding of this medical condition.

## Conclusions

Our study provides valuable insights into traumatic small bowel perforation, shedding light on its prevalence, patterns, mortality, and morbidity in a cohort of 40 participants, predominantly male (87.5%) within the age range of 25-50 years (50%). Most subjects presented a normal weight BMI (55%) and experienced blunt trauma injuries (75%), predominantly from motor vehicle accidents (57.5%). Abdominal tenderness was the most identified clinical sign (52.5%) while imaging mainly uncovered the presence of free intra-abdominal fluid. Initial laboratory findings showed a mean initial WBC level of 15.1 and an average initial amylase level of 77.2 U/L. Regarding interventions, 25% started with diagnostic laparoscopy, later converted to laparotomy, while 62.5% underwent direct exploratory laparotomy. The remaining 12.5% were managed non-surgically. The analysis revealed a positive correlation between gender, age, and morbidity. In contrast, a higher BMI indicated lower morbidity levels. Among the assessed mortality predictors, initial HCT level emerged as a significant association. The majority of patients with traumatic small bowel perforation who underwent surgical management had the perforated segment resected and anastomosed, representing 74.28% of the cases, while 25.72% underwent primary repair of the perforated segment. In terms of the intra-operative findings, single small bowel perforations were the most common type, accounting for 62.5% of the cases, followed by multiple small bowel perforations in 27.5% of the cases, and concomitant small bowel/perforation in 10% of the cases. Various injuries were observed in intra-abdominal organs, including spleen, kidney, liver, stomach, adrenal, and pancreas. The jejunum was the most common site of perforation, followed by the ileum, with specific distances from relevant anatomical landmarks reported. However, some cases lacked specific data on the site of perforation. These findings underscore the necessity for preventive strategies focusing on MVAs and identifying potential prognostic indicators. To enhance the understanding of the complex facets of traumatic bowel injuries, further research involving larger cohorts and prospective designs is duly recommended.
